# Predictive modeling of climate change impacts using Artificial Intelligence: a review for equitable governance and sustainable outcome

**DOI:** 10.1007/s11356-025-36356-w

**Published:** 2025-04-04

**Authors:** Kingsley Ukoba, Oluwatayo Racheal Onisuru, Tien-Chien Jen, Daniel M. Madyira, Kehinde O. Olatunji

**Affiliations:** 1 Department of Mechanical Engineering Science, Faculty of Engineering and the Built Environment, University of Johannesburg, Johannesburg, South Africa; 2 Cr Research Group, Department of Physics, Faculty of Sciences, University of Johannesburg, Johannesburg, South Africa; 3 Process Energy and Environmental Technology Station, Faculty of Engineering and the Built Environment, University of Johannesburg, Johannesburg, South Africa

**Keywords:** Predictive modeling, Climate change, Artificial Intelligence, Climate impact, Sustainable development

## Abstract

The accelerating pace of climate change poses unprecedented challenges to global ecosystems and human societies. In response, this study reviews the power of Artificial Intelligence (AI) to develop advanced predictive models for assessing the multifaceted impacts of climate change. The study used the PRISMA framework to find, assess, and combine research on using AI in predicting climate change impacts. Integrating AI techniques, such as machine learning algorithms and predictive analytics, into climate modeling provides a robust framework for understanding and projecting the complex dynamics associated with global climate change. These models exhibit a high capacity for data collection, analyzing intricate patterns and integration, including their relationships within the datasets. They enable quick and accurate predictions of future climate scenarios, scenarios testing, historical eventualities, their magnitude, and adaptation. However, challenging issues like data gaps, especially in interconnected systems such as the atmosphere, are associated. Also, AI insight translation into an actionable recommendation recognizable by the policymakers, including ethical usage, is an emerging concern. Therefore, further advances to circumvent these will include the integration of AI with physical models, developing hybrid models, and generating synthetic climatic datasets to enhance data quality and gaps. Also, AI tools are being developed to aid decision-making for policy integration. AI-based predictive modeling is restructuring and bringing reformative change to the understanding of and approach toward climatic change through AI model development. AI guarantees an unfailing plan and a resilient future with sustainable approaches that empower scientists, policymakers, and communities.

## Introduction

Climate change is one of our planet’s most pressing and complex challenges because of the existential threats toward every sphere of Earth’s systems, ecosystems, and human social circle. The Earth’s climate has undergone significant transformations over geological timescales through significant usage of fossils, radioactive dating, rock layers, and other geologic principles for certain date determination, but the current era is marked by unprecedented changes attributed to human activities (Peccerillo 2021). The combustion of fossil fuels, deforestation, industrial processes, and other anthropogenic factors, such as ecosystem alteration and extreme weather occurrences, have substantially increased greenhouse gas emissions, resulting in a warming planet and a cascade of interconnected climate impacts (Kumar et al. 2021), activated from complex-related web of consequential impacts.

The consequences of climate change are multifaceted and extend across various domains. Shifts in temperature patterns, altered precipitation regimes, rising sea levels, ecological imbalance, compelling modification of global inequality, and increased frequency and intensity of extreme weather events are just a few manifestations of this global phenomenon (Gahlawat and Lakra 2020). These changes pose significant threats to ecosystems, biodiversity, and human societies, impacting everything from agriculture and water resources to public health and infrastructure. Although efforts have been reported toward decarbonization for climate change mitigation, they are not without obstacles to be surmounted. This includes the utilization of wind power, nuclear energy, carbon pricing policy, and restructured environmentally friendly methodology by achieving environmental, economic, and energy security through an environmental taxation regulatory approach. However, the severity and frequency of these impacts continue to escalate, and there is an urgent need for accurate and reliable predictive models to guide mitigation and adaptation efforts (Alabdullah et al. 2023). Delaying in mitigation efforts poses an increase in cost, death risk, ecosystem collapse, global and economic instability, displacement and migration, and irreversible consequences.

Despite advances in climate science, predicting the specific impacts of climate change remains a formidable challenge (Peng et al. 2020) because of intertwined multifaceted issues that concern technological, political, social, and economic factors. The Earth’s climate is an intricate system with myriad interconnected variables, and predicting how changes in one component may reverberate across the entire system is complex. While valuable for understanding broad trends, traditional climate models, which are vital and essential tools for the perception, interpretation, and projection of the earth’s climate, often struggle with the granularity required to make precise predictions at regional or local scales (Chen et al. 2023; Machine et al. 2023). Modern computing methods can predict the commercial success of complex technologies.

Additionally, the inherent uncertainties in climate models stem from the dynamic nature of Earth’s systems (Deser et al. 2020), which include the atmospheric space, biosphere, hydrosphere, lithosphere, and cryosphere due to variation and continual flux within the components. Feedback loops, non-linearities, and the amplification of small initial changes present significant hurdles to accurate prediction. Variability in human behavior and socio-economic factors further complicates projections, as responses to climate change are shaped by myriad political, economic, and cultural considerations. Furthermore, the time scales involved in climate change make predictions challenging (Waldvogel et al. 2020) since the earth’s climate system process is influenced by immense and diverse temporal ranges, starting from second to millennia. While short-term weather forecasts ranging between a few hours and several days have become increasingly accurate because their process is based on real-time data and advanced computational models, long-term climate projections (prediction over extended periods within months and decades or centuries) have a higher degree of uncertainty. This temporal aspect is particularly crucial when addressing the need for timely and effective adaptation strategies.

Recognizing the limitations of traditional climate models, the integration of Artificial Intelligence (AI) has emerged as a promising avenue for advancing climate change impact prediction (Nishant et al. 2020). AI, particularly machine learning techniques, can analyze vast and complex datasets, identify intricate patterns, and discern relationships within data that may be challenging for traditional models to capture (Padhi et al. 2023). Machine learning algorithms, such as neural networks and ensemble methods, are adept at handling non-linear relationships and can adapt to changing patterns over time and discerning subtle trends that traditional climate models may overlook. The ability of AI models to learn from historical data and continuously update predictions as new information becomes available makes them well-suited for the dynamic nature of climate systems (Preethaa et al. 2023). It harnesses vast datasets encompassing meteorological, oceanographic, and environmental variables to train AI models.

The application of AI in climate modeling involves leveraging extensive datasets encompassing meteorological, oceanographic, and environmental variables (Sun 2023). This allows for a more comprehensive understanding of the intricate interactions contributing to climate change impacts. AI also facilitates improved prediction accuracy, enabling scientists and policymakers to anticipate changes at finer spatial and temporal scales (Nandgude et al. 2023). It augments traditional climate models by offering a complementary approach that captures Earth’s systems’ complexity and nuance (Chen et al. 2023; Machine et al. 2023). This integration can enhance the precision of climate change impact assessments, providing valuable insights for devising effective mitigation and adaptation strategies. On the other hand, another feature exhibited by AI is its potential to solve and prevent multifactorial techno-economic problems through prediction significantly, equipment failure early detection, maintenance, and optimization, thereby enhancing productivity, economic efficiency, cost-effectiveness, sustainability, and equipment lifecycle extension (Kliestik et al. 2023). The outcomes of this study hold significant implications for policy formulation, resource allocation, and sustainable development planning. Integrating AI in predictive climate modeling advances our understanding of climate change impacts. It empowers decision-makers with actionable insights to navigate the complex challenges a rapidly changing climate poses. As the urgency to address climate change intensifies, the synergy between AI and climate science emerges as a promising avenue for forging a resilient and sustainable future.

## Literature review

Traditional climate modeling methods have served as the backbone of climate science, providing valuable insights into Earth’s complex climate systems (Fan et al. 2021). These models, often based on physical principles, simulate the interactions between various components of the Earth’s atmosphere, oceans, land surface, and ice. General Circulation Models (GCMs) are widely used to project long-term climate trends and are crucial for understanding the broader implications of climate change on a global scale (Wu et al. 2020).

While traditional models offer fundamental insights, they face challenges in capturing fine-scale details and addressing uncertainties associated with complex climate interactions. Spatial and temporal resolutions are often limited, hindering their ability to make localized predictions. Additionally, the reliance on deterministic formulations may overlook certain non-linear and feedback-driven processes, contributing to a gap in accurately predicting regional and localized climate impacts.

The limitations of traditional climate models become apparent when confronted with the intricacies of climate change impacts (Jain et al. 2023). These models are challenged by Earth’s systems’ dynamic and interconnected nature, where subtle changes in one component can lead to cascading effects throughout the entire climate system. The non-linearities, feedback loops, and uncertainties associated with complex climate processes pose significant challenges to accurately predicting climate change’s diverse and evolving impacts (Ripple et al. 2023).

Moreover, traditional models may struggle to incorporate socio-economic factors and human behaviors, crucial in shaping vulnerability and resilience to climate impacts (Cianconi et al. 2021). The need for more granular, adaptable, and interpretable models is evident, as the consequences of climate change manifest at regional and local levels, impacting ecosystems, communities, and economies differently.

The emergence of Artificial Intelligence (AI) has introduced a paradigm shift in climate science, offering a complementary approach to traditional modeling methods. AI, particularly machine learning, has demonstrated the capacity to analyze large and complex datasets, identify patterns, and make predictions with a level of accuracy and adaptability that traditional models may struggle to achieve (Padhi et al. 2023). Machine learning algorithms, such as neural networks, decision trees, and ensemble methods, can handle non-linear relationships and adapt to changing patterns over time. This adaptability makes AI models well-suited for climate systems’ dynamic and evolving nature. Moreover, AI models can learn from historical data, capturing intricate relationships and improving predictive capabilities over time.

### Previous studies utilizing AI for climate impact prediction

Several pioneering studies have explored the application of AI in predictive modeling of climate change impacts, providing valuable insights and paving the way for further research (Al-Sai et al. 2022). For example, researchers have utilized machine learning techniques to predict temperature and precipitation patterns with higher accuracy at regional and local scales (Ahmed et al. 2020; Wang et al. 2021). Ensemble models, combining the strengths of multiple algorithms, have been employed to enhance the robustness of predictions and quantify uncertainties. AI has also been instrumental in assessing the impacts of climate change on specific ecosystems, such as coral reefs, forests, and agricultural landscapes (Owens 2020). These studies leverage AI to analyze complex interactions between climate variables and ecological responses, contributing to a more nuanced understanding of how climate change influences biodiversity and ecosystem services. Furthermore, the interpretability of AI models has been a focal point, with efforts to make predictions more transparent and accessible to a broader audience. Collaborations between climate scientists and AI experts have been instrumental in refining models and addressing model interpretability challenges, promoting interdisciplinary approaches to tackling climate change (Bibri et al. 2024).

In summary, the literature review underscores the limitations of traditional climate modeling methods and the potential of AI to bridge existing gaps. The use of AI in climate science opens new avenues for refining predictive models, improving accuracy, and enhancing our understanding of the diverse impacts of climate change (Singh and Goyal 2023). The subsequent sections of this paper will delve into the methodologies employed in predictive modeling using AI, the challenges faced, and the implications for policy formulation and sustainable development. By incorporating AI-driven predictions, the study enhances the precision and reliability of climate impact assessments, thereby assisting policymakers, researchers, and stakeholders in devising adaptive strategies. Furthermore, the research delves into the interpretability of AI models, aiming to enhance the transparency and understanding of the predictions generated.

## Methodology

**Fig. 1:**
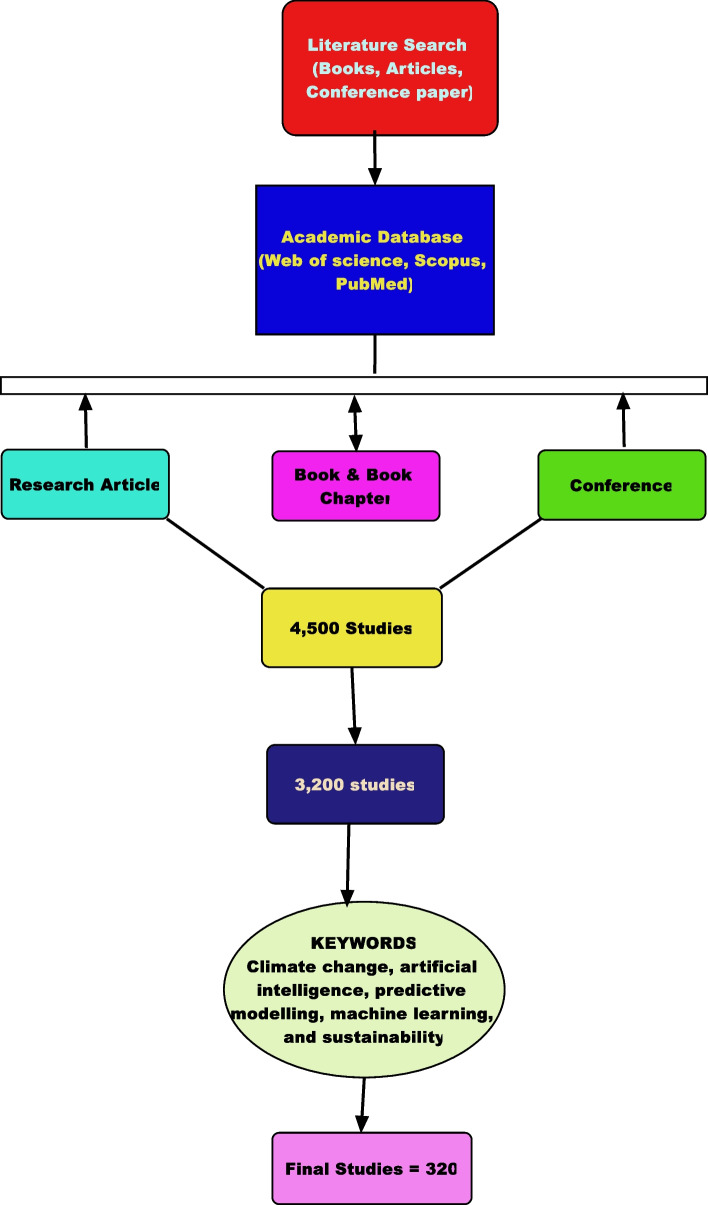
PRISMA flowchart for AI in climate change review

This flowchart, shown in Fig. 1, shows the PRISMA process for picking studies in this review. It details the steps from searching databases to including studies in the final analysis. The study used the PRISMA framework to find, assess, and combine research on using AI in predicting climate change impacts. The goal is to keep the process clear and repeatable. The study searched for peer-reviewed articles, conference papers, and books in several academic databases like Web of Science, Scopus, and PubMed. It used keywords like climate change, Artificial Intelligence, predictive modeling, machine learning, and sustainability. The study sets clear rules for including studies. Only studies about AI’s role in climate change prediction, fair outcomes, and sustainable solutions were chosen. Thereafter, it excluded anything not published in English, neither peer-reviewed nor related to AI and climate change. The initial search found 4500 studies; upon removing duplicates, 3200 studies were left. Screening titles and abstracts cut this down to 1200. Full-text reviews led to 320 articles that met our criteria.

In data extraction, the study looked at the AI methods used, how accurate the predictions were, the datasets, the geographical focus, and governance issues. The study used a checklist to judge the quality of studies, checking their relevance and reliability. Any disagreements among reviewers were solved through discussion. The thematic analysis was used to combine the data collected. This analysis looked at important AI methods like machine learning and deep learning, the impact areas like precipitation and temperature, and how they relate to fair governance and sustainability.

Figure 1 shows the flowchart of the PRISMA process for picking studies for this study. It walks through the steps starting from the first database search all the way to which studies that was included in the final summary.

The foundation of predictive modeling for climate change impacts using Artificial Intelligence (AI) lies in acquiring comprehensive, high-quality datasets (Sun et al. 2022). Incorporating a diverse range of data, including meteorological, oceanographic, and environmental variables, helps ensure a holistic understanding of the complex interactions driving climate change (Wells et al. 2020). Meteorological data encompassing temperature, precipitation, wind patterns, and atmospheric pressure are vital in capturing atmospheric dynamics. Oceanographic variables such as sea surface temperatures, ocean currents, and salinity contribute crucial information about the state of the world’s oceans. Environmental data, including land use, vegetation cover, and anthropogenic activities, is included to account for the diverse factors influencing climate systems (Yang et al. 2021). Data sources are carefully selected based on their reliability, spatial and temporal resolutions, and global coverage (Davison et al. 2021). Satellite observations, ground-based monitoring stations, and climate model outputs contribute to the dataset, ensuring a combination of observational and simulated data (Ma et al. 2021). The data quality is assessed through rigorous validation processes, and efforts are made to address potential biases and inaccuracies.

The pre-processing phase is critical in refining the raw data, enhancing its quality, and preparing it for effective utilization in AI models (Maharana et al. 2022). Raw datasets often contain missing or erroneous values, which can compromise the integrity of the predictive models. Robust data cleaning procedures are implemented to identify and address missing data points, employing techniques such as imputation or, when necessary, excluding incomplete records (Alruhaymi et al. 2021). This step is crucial to ensure the reliability of input data for subsequent analyses. In dealing with multidimensional datasets, feature selection and extraction become vital to identify the most relevant variables influencing climate change impacts. Advanced statistical methods and machine learning techniques are applied to discern the key features contributing to the model’s predictive power (Zhong et al. 2021). Dimensionality reduction techniques, such as Principal Component Analysis (PCA), may be utilized to streamline the dataset while preserving critical information (Chhikara et al. 2022). The selection of AI algorithms plays a pivotal role in the success of predictive modeling for climate change impacts. The chosen algorithms must be able to handle the complexity of climate data, capture non-linear relationships, and adapt to changing patterns over time (Elbeltagi et al. 2023).

Various machine learning techniques are considered, including neural networks, decision trees, and ensemble methods. Neural networks, inspired by the structure of the human brain, are adept at capturing intricate patterns and relationships within the data (Hasson et al. 2020). Decision trees offer transparency in decision-making processes, making them valuable for interpretability. Ensemble methods, such as Random Forests or Gradient Boosting, combine multiple models to enhance predictive accuracy and mitigate overfitting (González et al. 2020). The selection of specific algorithms is guided by the nature of the climate data and the objectives of the predictive modeling (Jebli et al. 2021). For instance, neural networks may be preferred when dealing with complex, non-linear relationships, while ensemble methods provide robustness in handling uncertainties. The rationale behind choosing a particular algorithm is grounded in its suitability for the dataset’s characteristics, the interpretability of results, and the computational efficiency required for large-scale climate modeling (Hissou et al. 2023).

### AI model development

Training AI models involves carefully partitioning the dataset into three distinct subsets: training, validation, and testing (Castiglioni et al. 2021). The training set serves as the foundation for the model to learn the underlying patterns and relationships within the data. The validation set, separate from the training data, is utilized to fine-tune the model’s hyperparameters and prevent overfitting (Elgeldawi et al. 2021). Finally, the testing set evaluates the model’s generalization performance on unseen data, providing a realistic measure of its predictive capabilities. The partitioning ensures that the model is exposed to diverse patterns in the training data, refined using the validation set, and rigorously tested for its robustness and accuracy on the testing set (Akbulut et al. 2023). This division prevents the model from memorizing the training data (overfitting) and ensures it can make reliable predictions on new, unseen data.

Hyperparameters are critical configuration settings that influence the learning process of AI models. Tuning hyperparameters involves adjusting these settings to optimize the model’s performance. Common hyperparameters include learning rates, batch sizes, and the depth of neural networks. To achieve the most effective model, an iterative process of adjusting hyperparameters based on the performance of the validation set is conducted (Shahhosseini et al. 2022). Techniques such as grid search or randomized search are often employed to explore the hyperparameter space systematically. This iterative tuning process ensures that the AI model converges to an optimal configuration, balancing the need for complexity to capture intricate patterns with the risk of overfitting the training data.

**Fig. 2:**
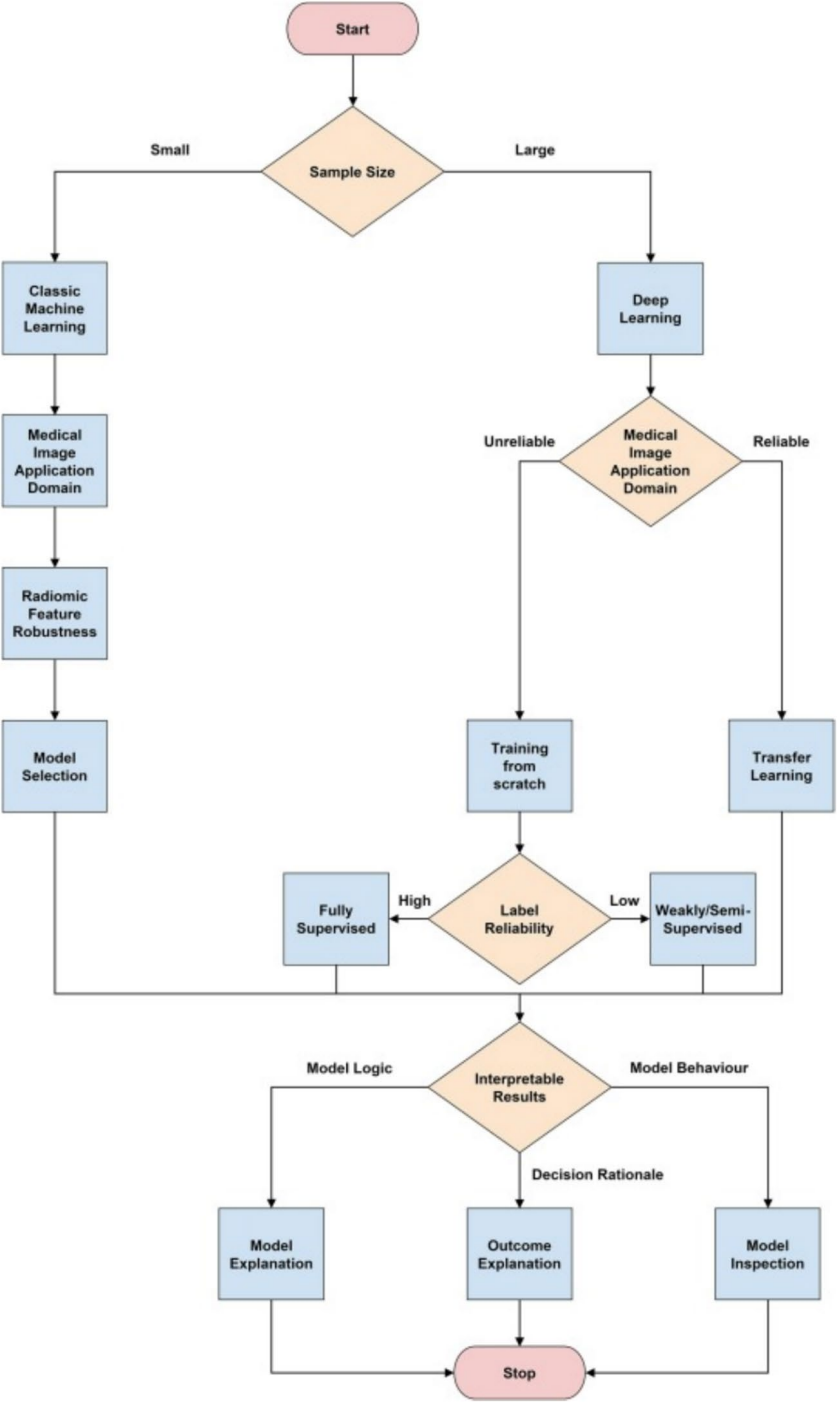
Flow diagram for the design choices in Artificial Intelligence model development (Castiglioni et al. 2021)

The evaluation of AI models involves the application of metrics to quantify their performance, as explained in Fig. 2 (Castiglioni et al. 2021). Various metrics are employed to assess the model’s predictive capabilities, depending on the nature of the climate change impact predictions (Dikshit and Pradhan 2021).

Mean Squared Error (MSE) is a standard metric for regression tasks, measuring the average squared difference between predicted and actual values (lowast and Kumari 2020). Lower MSE values indicate better predictive accuracy. For classification tasks, accuracy represents the overall correctness of predictions. At the same time, precision focuses on the accuracy of positive predictions, providing insights into the model’s ability to identify specific climate change impacts correctly. Area Under the Receiver Operating Characteristic (ROC) Curve (AUC-ROC) is particularly relevant for binary classification problems; AUC-ROC assesses the trade-off between true positive and false positive rates, providing a comprehensive view of model performance across different thresholds (Jaskowiak et al. 2020). The selection of metrics depends on the specific objectives of the climate change impact prediction task and the dataset’s characteristics.

**Fig. 3:**
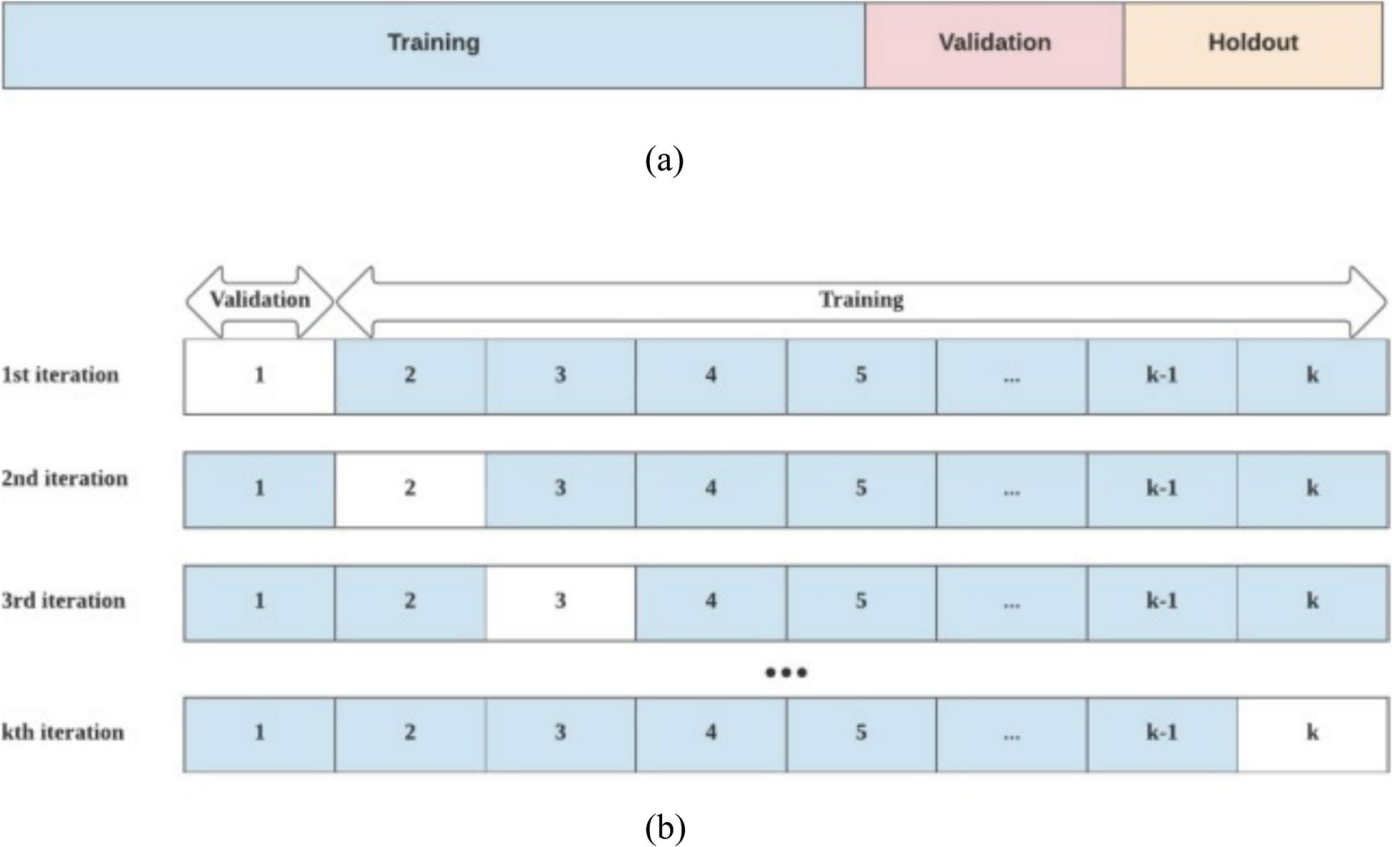
Diagram of k-fold forward cross-validation (Xiong et al. 2020)

Cross-validation techniques are employed to ensure the robustness and generalization of the AI model. Cross-validation involves splitting the dataset into multiple folds, training the model on different subsets, and evaluating its performance on the remaining data, as illustrated in Fig. 3 (Xiong et al. 2020).

Standard cross-validation methods include k-fold cross-validation, where the dataset is divided into k subsets, and each fold is used as a testing set while the remaining folds constitute the training set in a rotating manner (Rauber et al. 2021). Cross-validation helps assess how well the model generalizes to different subsets of the data, providing a more reliable estimate of its performance on unseen data (Bates et al. 2023). This step is crucial for avoiding biases that might arise from a specific data partition.

In summary, developing AI models for predicting climate change impacts involves a meticulous training, validation, and evaluation process. By strategically dividing datasets, tuning hyperparameters, and employing appropriate evaluation metrics and cross-validation techniques, researchers aim to create robust models that can contribute valuable insights to understanding complex climate dynamics and support informed decision-making for mitigation and adaptation strategies.

### Predictive modeling of climate change impacts

Climate change, driven by human-induced factors such as greenhouse gas emissions, poses profound challenges to the stability of Earth’s systems (Wang et al. 2023). Predictive modeling, particularly through the integration of Artificial Intelligence (AI), offers a robust approach to understanding and anticipating the diverse impacts of this global phenomenon (How and Chan 2020). Predicting shifts in temperature patterns is a core component of climate change impact modeling. AI models, with their ability to discern complex patterns, enhance the accuracy of temperature predictions at both global and regional scales (Alizadeh 2022). To project future temperature trends, these models consider historical temperature data, atmospheric conditions, and greenhouse gas concentrations. This predictive capability is crucial for anticipating changes in growing seasons, thermal stress on ecosystems, and species’ geographic redistribution.

By incorporating AI into climate models, researchers can identify non-linear relationships and feedback mechanisms contributing to temperature fluctuations (Ahmed et al. 2020). This enables a more nuanced understanding of how temperature changes may vary across different regions, allowing for targeted adaptation strategies. Changes in precipitation patterns, including alterations in rainfall intensity, frequency, and distribution, represent another significant aspect of climate change impact modeling. Moreover, fossil fuels, which are part of the fundamental drivers of anthropogenic play a major role in climate change as it directly influences global energy consumption patterns. In industrial processes, energy production, and transportation, the decreased price of fossil fuel encourages continual reliance, higher demand, and consumption, which generates increased carbon emissions. Also, the higher price of fossil likewise stimulates alternative energy solution investment. Although the prediction complexity of fossil is associated with higher volatility of oil prices, it has been reported that artificial neural network-Long Short-Term Memory (LSTM) has the capability for oil price prediction*.* AI models excel in analyzing large and complex datasets related to atmospheric conditions, oceanic influences, and land-use changes, allowing for more accurate predictions of precipitation changes (Salcedo-Sanz et al. 2020). AI’s capacity to capture non-linear relationships is particularly beneficial when assessing the complex interplay between temperature and precipitation. These models contribute to identifying areas vulnerable to droughts, floods, or shifts in seasonal precipitation, providing critical information for water resource management, agriculture, and disaster preparedness.

Sea level rise, a consequence of melting glaciers and ice sheets and the thermal expansion of seawater, is a direct consequence of global warming (Shukla et al. 2021). AI models aid in predicting the extent and pace of sea level rise by integrating data on ice melt, ocean currents, and thermal expansion. Satellite observations and climate model outputs contribute to the datasets that train these models. The predictive capability of AI facilitates more accurate assessments of coastal vulnerability, enabling communities and policymakers to plan for sea level rise impacts on infrastructure, habitats, and human settlements (Mokhtar et al. 2023). This information is vital for implementing adaptive measures and designing resilient coastal zones. The frequency and intensity of extreme weather events, including hurricanes, droughts, heat waves, and storms, are escalating due to climate change (Aghakouchak et al. 2020). AI models enhance the prediction of these events by analyzing historical weather data, oceanic conditions, and atmospheric dynamics. The adaptability of machine learning algorithms allows for identifying subtle patterns indicative of potential extreme events.

AI’s real-time processing of vast data sets contributes to developing early warning systems and improving preparedness and response capabilities. By predicting the likelihood and severity of extreme weather events, communities can implement timely evacuation plans, reinforce infrastructure, and reduce the risks associated with climate-induced disasters. Climate change impacts extend beyond individual events, leading to cascading effects on ecosystems and human communities (Upadhyay and Upadhyay 2020). By considering the interconnectedness of various factors, AI models contribute to a more holistic understanding of these cascading effects. The models incorporate data on temperature, precipitation, sea level rise, and extreme events to assess how changes in one component may trigger a chain reaction of consequences. In ecosystems, predictive modeling through AI helps identify vulnerable species, assess habitat suitability, and anticipate shifts in biodiversity. Understanding the cascading effects on human communities involves analyzing socio-economic factors, population dynamics, and infrastructure vulnerabilities in the face of climate-induced changes.

AI’s predictive modeling of climate change impacts is a pivotal tool for addressing the multifaceted challenges posed by a warming planet. By focusing on temperature shifts, altered precipitation patterns, sea level rise, extreme weather events, and cascading effects on ecosystems and human communities, these models contribute invaluable insights that empower societies to develop proactive strategies for climate change adaptation and mitigation (Danylchuk et al. 2023).

Recent advancements and emerging trends in AI applications and climate studies have shown that weather can be forecasted accurately with AI, which will assist in climate change adaptation and disaster management. Meteorologists can analyze huge amounts of weather data in real-time using deep learning algorithms to improve the accuracy of long- and short-term weather prediction. This development has led to more effective preparations for extreme weather conditions and assisted in reducing their impact on vulnerable communities (Lewis et al. 2024). To improve climate models, a framework that uses physically constrained generative adversarial networks was developed to increase the accuracy of precipitation patterns simulation in the Earth system models (ESMs). It was reported that the developed model outperformed the existing models by correcting the local distributions and enhancing spatial patterns significantly, especially daily precipitation intermittency. One of the challenges of ESMs is the double-peaked Intertropical Convergence Zone, which was removed successfully with this approach (Hess et al. 2022). The study highlighted the strength of utilizing generative adversarial networks, and AI approaches to improve the realism and accuracy of climate models. With accurate precipitation patterns, scientists can provide more reliable predictions and projections to assist in disaster management and climate change adaptation approaches. One of the crucial strategies to mitigate climate change is the transition to renewable energy origins. AI is crucial to renewable energy optimization and improved efficiency. Machine learning models can analyze weather patterns, energy demand, and grid conditions to forecast renewable energy production and its optimization into the power grid. Optimization and analyses of renewable energy improve its reliability and stability, which will assist in reducing the reliance on fossil fuels (Ukoba et al. 2024). Table 1 presents the application of some AI models in climate studies with their strengths and limitations. It can be observed that these models still have some limitations, which require further studies for better and more efficient modeling in climate science.

**Table 1:** Different AI applications in climate impacts with their strengths and limitations

*S*/*N*	AI models	Applications	Strength	Limitations	Ref
1	Recurrent Neural Networks (RNNs)	Flood prediction using rainfall data	- Suitable for sequential data processes like time series prediction and natural language processing - It can learn long-term dependencies in data	- Times series prediction (e.g., extreme weather conditions prediction) - Natural language processing (e.g., information extraction from environmental reports)	(Ren et al. 2020)
2		Prediction of river flow level			(Liu et al. 2020)
3	Decision Trees	Prediction of water quality in rivers	- It is relatively simple to train and interpret - It can be used for a variety of data types	- Water quality monitoring - Detection and monitoring of deforestation - Air quality monitoring	(Nouraki et al. 2021)
4		Assessment of water quality			(Bui et al. 2020; Nasir et al. 2022)
5	Convolutional Neural Networks (CNNs)	Monitoring of coastal erosion	- It can learn complex patterns from images without explicit programming - Most suitable for image classification and other assignments that involve spatial data	- Image classification (e.g., wildlife identification, deforestation detection)	(Scardino et al. 2022)
6		Monitoring coral reef health through underwater images			(Burns et al. 2022)
7	Support Vector Machines (SVMs)	Air quality monitoring	- Suitable for varying data types, including text and images - It can process high-dimensional data - It can assimilate complex relationships within variables	- Natural language processing (e.g., information extraction from environmental reports) - Time series prediction (e.g., forecasting extreme weather conditions) - Image classification (e.g., wildlife identification, deforestation detection)	(Castelli et al. 2020; Leong et al. 2020)
8		Prediction of harmful algal biomass in lakes			(Henrique et al. 2021)
9	Random Forests	Modeling air quality index in urban areas	- Have better strength for overfitting than individual decision trees - An ensemble learning technique that integrates several decision trees' predictions	- Image classification (e.g., wildlife identification, deforestation detection) - Natural language processing (e.g., information extraction from environmental reports) - Time series prediction (e.g., forecasting extreme weather conditions)	(Alsaber et al. 2021)
10		Prediction of forest fire			(Decastro et al. 2022; Pham et al. 2020)
11	Hybrid models	Combination of RNNs and CNNs to predict air pollution	- Combined the strength of deep learning and machine learning - More robust and accurate than individual models	Not suitable for environmental task monitoring that requires high accuracy and robustness	(Tsokov et al. 2022)
		A combination of CNNs and RNNs is used to predict urban heat islands			(Li and Zheng 2023)

### Interpretability and transparency in AI model development

**Fig. 4:**
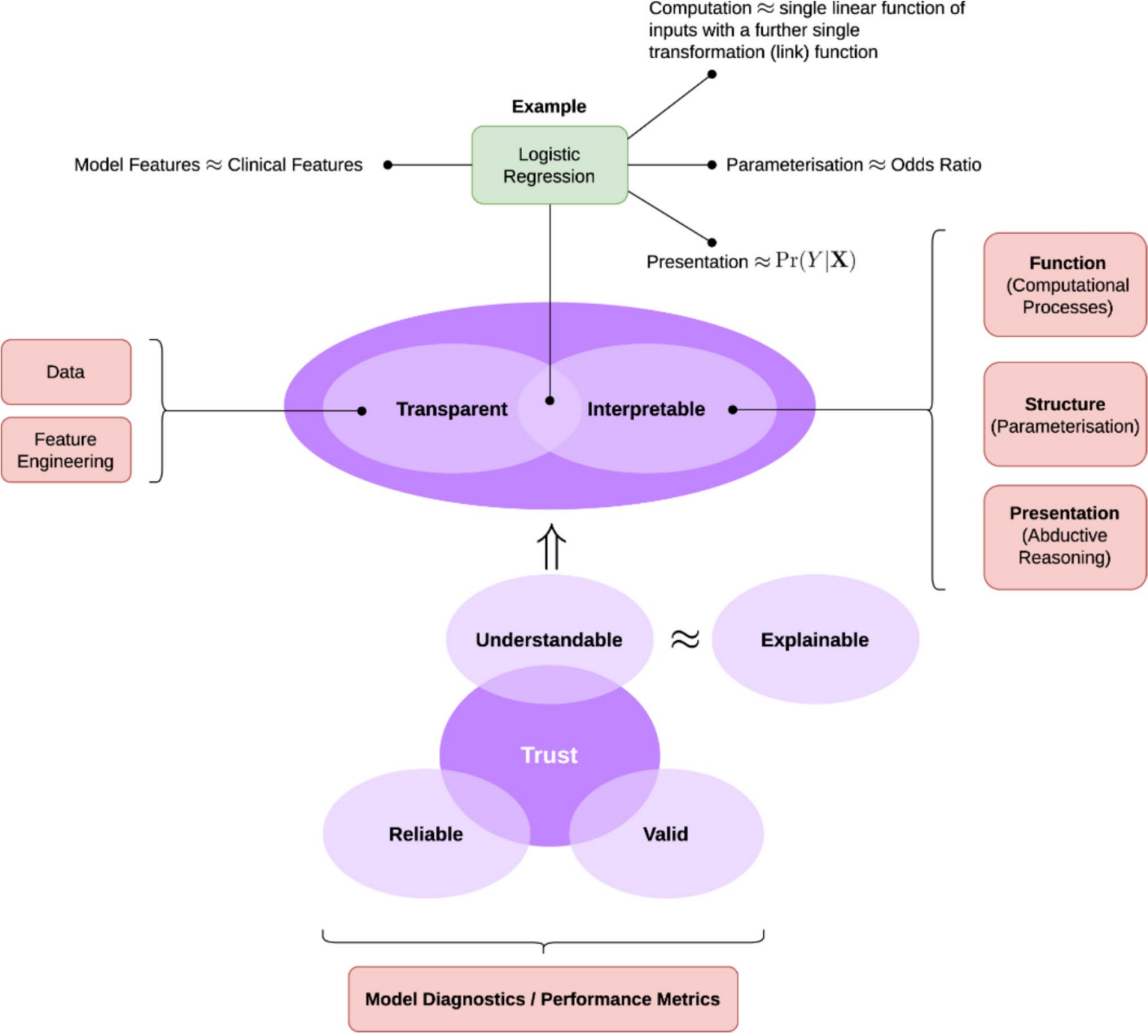
The transparency and interpretability for understandable models (TIFU) (Joyce et al. 2023)

Interpretability and transparency are crucial aspects of AI model development, particularly in predicting climate change impacts (Stiglic et al. 2020). As these models play an increasingly pivotal role in shaping environmental policies and guiding adaptive strategies, understanding and trust in their predictions are paramount, as presented in Fig. 4 (Joyce et al. 2023). This section explores the methods used to enhance interpretability, the collaborative efforts between climate scientists, AI experts, and stakeholders, and the measures taken to ensure transparency in predictions. Understanding which features or variables significantly influence the model’s predictions is fundamental for interpretability. Feature importance analysis, often employed in ensemble methods like Random Forests, helps identify the most impactful variables in the climate change impact prediction model (Naseri et al. 2021). This information is vital for climate scientists and policymakers to comprehend the critical drivers behind projected changes. Explainable AI techniques aim to demystify complex models, making their decision-making processes more transparent. Methods such as LIME (Local Interpretable Model-agnostic Explanations) and SHAP (SHapley Additive exPlanations) generate interpretable explanations for individual predictions, shedding light on how the model arrives at specific outcomes (Dwivedi et al. 2023). Integrating XAI techniques ensures that the intricate relationships within the model are accessible and understandable. Visual representations of model outputs, such as heat maps or interactive graphs, provide an intuitive means of conveying complex information. Climate scientists can use these tools to communicate the model’s predictions to a broader audience, including policymakers and the public, fostering a shared understanding of the projected impacts.

Effective collaboration between climate scientists and AI experts is essential for developing models that align with both the scientific rigor of climate research and the technical sophistication of AI methodologies (McIntosh et al. 2023). Interdisciplinary teams combine domain knowledge from climate science and expertise in machine learning, ensuring that the predictive models accurately reflect the complex dynamics of the Earth’s climate. In addition to the collaboration between scientists and AI experts, the inclusion of stakeholders, such as policymakers, local communities, and environmental organizations, is vital. Stakeholders bring contextual insights, data on socio-economic factors, and perspectives on the real-world implications of climate change impacts (Petutschnig et al. 2023). Engaging with these stakeholders throughout the model development process ensures that the models address relevant concerns and contribute to actionable solutions. Collaborative efforts should extend beyond technical aspects to include ethical considerations. Discussions on data privacy, model biases, and the ethical use of AI in climate modeling are essential for building stakeholder trust. Ensuring transparency about how data is sourced, processed, and utilized is crucial to ethical AI development. Transparency in data sources and methodologies is fundamental for establishing trust in AI predictions. Adopting open data practices, where datasets and model codes are publicly accessible, allows for scrutiny and validation by the scientific community, contributing to the transparency of the entire modeling process. Comprehensive documentation of the AI model’s architecture, training process, and validation results enhances transparency. This documentation is a reference for climate scientists, AI experts, and stakeholders to understand the model’s intricacies and the reasoning behind its predictions. Clear and accessible reporting is essential for transparent communication of model outputs to a broader audience. Climate change predictions inherently come with uncertainties, and AI models are no exception. Transparently communicating these uncertainties, whether from data limitations, model assumptions, or inherent variability in climate systems, is crucial for providing a realistic portrayal of the predictions (Rounsevell et al. 2021). Techniques such as probabilistic modeling can be utilized to quantify and convey uncertainties in AI predictions.

Enhancing interpretability and transparency in AI model development for climate change impact prediction is a technical and ethical imperative. By employing methods for interpretability, fostering collaboration between diverse experts, and implementing measures to ensure transparency, the resulting models can contribute to a shared understanding of climate change impacts and support informed decision-making for a sustainable future.

Carbon sequestration methods aim to capture and store carbon dioxide (CO_2_) to mitigate climate change. Two notable approaches discussed in the context include carbon capture in industrial processes and bioenergy-based solutions (Maroušek et al. 2023). The first approach leverages industrial by-products to reduce carbon emissions. Waste refining for cement substitutes, such as the utilization of fly ash or slag, decreases reliance on traditional cement production, a significant CO_2_ emitter. These substitutes integrate captured CO_2_ into alternative materials, achieving sequestration while providing cost-effective construction solutions. This method combines waste management with carbon reduction, exemplifying sustainable industrial practices.

The second approach focuses on bioenergy-based carbon sequestration, specifically using solid biofuels supported by subsidies, as analyzed in the Czech Republic. Biomass-derived fuels, like wood pellets and agricultural residues, act as renewable energy sources while sequestering carbon through natural photosynthesis. Subsidies incentivize the cultivation of bioenergy crops and utilization of agricultural waste, promoting carbon–neutral energy cycles. The combustion of these biofuels, combined with carbon capture and storage (CCS) technologies, further enhances sequestration efficiency. Both methods highlight the importance of integrating economic incentives and innovative technologies to maximize the potential of carbon sequestration in combating global CO_2_ emissions.

The predictive models can be used to understand how climate change affects things like temperature changes, rainfall patterns, sea level rise, and extreme weather. These models also help to see how these changes impact people and the environment. Working together is a big part of this. Climate scientists, AI experts, and others can team up to improve these models. This helps ensure the models are useful for planning ways to deal with climate change. Bringing ideas from economics can also help. By looking at costs and benefits, as well as how people make choices, climate models can be improved even better. Thus, climate change can be connected to how people and resources are managed. Some of these include future emission estimation based on population growth, increased energy usage, advancement in technological growth, and analysis of industrial and individual responses to policies. The benefits include scenario testing and global teamwork for equitable distribution efforts toward mitigation. Integrated Assessment Models (IAMs) such as the Dynamic Integrated Climate-Economy (DICE) and Framework for Uncertainty, Negotiation, and Distribution (FUND) models serve as tools and direct approaches in economics models. Climate policy simulation with the economy using Computable General Equilibrium (CGE) models is conducted. AI-driven climate models have been applied to different climate sciences and have assisted in making crucial decisions on natural disasters that may result from improper climate management. Table 2 illustrates some of the applied models’ performance and influence on decision-making.

**Table 2:** AI-driven climate models application and impacts on decision-making

*S*/*N*	Climate actions	Models used	Performance	Impacts on decision-making	Ref
1	Monthly rainfall	ANN and ANFIS	ANFIS showed higher accuracy	It aided the meteorological stations in weather prediction and planning	(Abebe and Endalie 2023)
2	Pollutant and particle levels	Support Vector Regression (SVR)	Pollutants like CO, O_3_, and SO_2_ were predicted at 94.1% accuracy	The Environmental Protection Agency was able to plan with the data	(Castelli et al. 2020)
3	Earthquake	ANN and Random Forest	ANN model produced better earthquake depth, acceleration, and velocity accuracy	It is used in planning for the natural disaster and its influence on the environment	(Essam et al. 2021)
4	Origin of trace pollutants	Long Short-Term Memory Network (LSTM)	It provides high prediction accuracy and traces the significant sources of the pollutant	The result was used to control pollutant discharge and enhance the water quality	(Wang et al. 2019)
5	Forest fire	Classification and Regression Tree (CART)	The slope showed a substantial influence on forest fire occurrence	It assists in preparation for the forest fire occurrence	(Piao et al. 2022)
6	Projection of flue gas from waste-to-energy	ANN	The mean square error of the model validation constraints is between 0.003 and 0.19	Findings from the study were used to make decisions in renewable energy planning	(Ma et al. 2023)
7	Quality of water for drinking	SVM and Logistic Regression	The models predicted the water quality at 98 and 92%, respectively	The data from this study assisted in expanding the scope of the task	(Panigrahi et al. 2023)
8	Changes in water level	Multi-layer Perception Neural Network (MLP-NN)	The models accurately predicted changes in water level with running time	It was assisted in planning for flooding	(Nur Adli Zakaria et al. 2021)

### Comparison of this study with existing literature

The findings of this study align with and deviate from existing literature in several ways, influenced by methodological approaches, data challenges, AI models, applications, and governance considerations. The review aligns with studies such as Aghakouchak et al. (2020) and Ahmed et al. (2020) by emphasizing ensemble machine learning models for climate prediction, highlighting techniques like bagging, boosting, and multi-model integrations to enhance accuracy. Like Alizadeh (2022), the review discusses challenges in modeling extreme events due to data variability, advocating robust data integration strategies. However, it places less emphasis on explainability than works like Dikshit and Pradhan (2021), which stress interpretability in AI models for drought prediction. Furthermore, the review sufficiently explores hybrid models combining federated learning and machine learning compared to that of Akbulut et al. (2023), limiting insights into privacy-preserving modeling. These deviations may result from differences in data accessibility, computational resources, and regional focus, as federated learning is more prevalent in privacy-sensitive regions.

In addressing data challenges, the review and Alruhaymi et al. (2021) highlight the impact of missing data and the importance of imputation techniques, consistent with Salcedo-Sanz et al. (2020), which emphasizes the fusion of satellite, sensor, and historical data. However, it offers limited discussion on integrating human dimensions into climate models compared to Hurlbert et al. (2019) and Davison et al. (2021), which focus on socioeconomic and land-use data. The emphasis on biophysical over socioeconomic data in the reviewed study could stem from its narrower research scope and the complexity of modeling anthropogenic factors alongside physical processes. The review identifies ensemble methods like random forests and gradient boosting as top-performing techniques, resonating with González et al. (2020) and Bates et al. (2023). However, it does not explicitly discuss interpretability solutions like SHAP or LIME, as Dwivedi et al. (2023) emphasized, which are crucial for stakeholder acceptance. Additionally, the role of deep neural networks in downscaling climate variables, highlighted by Wang et al. (2021), is underexplored. These deviations may arise from prioritizing performance metrics like accuracy over transparency and practical applicability, potentially due to differing research objectives.

The review aligns with Yigitcanlar et al. (2021) in discussing AI’s role in smart city solutions for mitigating climate impacts, focusing on real-time monitoring and adaptive systems, and it echoes Jain et al. (2023) by exploring AI’s potential in protecting infrastructure. However, it diverges from Ripple et al. (2023), which highlights feedback loops and tipping points in climate models, focusing more on steady-state predictions. This could result from its focus on near-term governance applications rather than long-term systemic changes, influenced by its aim to support policy over scientific advancements.

The review emphasizes the importance of equitable AI governance frameworks to mitigate biases in predictive modeling, consistent with Nishant et al. (2020). It also advocates participatory decision-making processes informed by AI-driven insights, aligning with Kulkov et al. (2023). However, it underemphasizes the socio-economic trade-offs of AI applications explored by Hissou et al. (2023), potentially missing key barriers to equitable implementation. The focus on technical aspects over socio-economic considerations may result from disciplinary boundaries or a narrower scope aimed at technological audiences. Integrating AI with socioeconomic and land-use data, as discussed by Davison et al. (2021), would strengthen the reviewed study’s outcomes. Incorporating interpretability frameworks like SHAP or LIME would enhance stakeholder trust, addressing gaps identified by Dwivedi et al. (2023). Leveraging federated learning approaches from Akbulut et al. (2023) could address privacy concerns while improving model robustness. Addressing these deviations could improve alignment with contemporary advancements, fostering both technical precision and socio-economic relevance.

## Implications for policy and decision-making

Using AI, predictive modeling of climate change impacts brings transformative implications for policy formulation, resource allocation, and sustainable development planning (Yigitcanlar et al. 2021). As the urgency to address climate change intensifies, integrating AI-driven predictions into decision-making processes becomes essential for developing adaptive strategies that mitigate environmental risks and promote long-term sustainability.

Different policies have been formulated locally and internationally to solve the challenges of climate change’s impact on the world. These policies differ from country to country depending on the geographical location, available resources, and some other essential factors. Table 3 illustrates some climate change mitigation policies, their limitations, and how AI applications can improve their implementation. It was noticed from the table that some of these policies have not fully achieved their set target because of some challenges. The application of AI can improve the impact of these policies that are essential to climate change strategy policies (Corbera et al. 2019).

**Table 3:** Climate change policies, limitations, and the expected role of AI

*S*/*N*	Existing climate change policies	Limitations	The role of AI is to improve the implementation
1	Clean and affordable energy	Biofuel production to lower the dependency on fossil fuels that damage the climate is not always economical	Using AI to predict the biofuel yield from the feedstock before production will make the process more economical and acceptable
2	Sustainable waste management	Waste management is still a major challenge in several countries because of poor planning and implementation	Waste generation prediction using AI models could assist the policymakers in formulating policy that will be sufficient to manage future waste generation instead of waiting until it becomes an environmental disaster
3	Decarbonization	Like solar energy, green energy is still inefficient in reducing carbon emissions	Adopting GIS to select solar farm locations instead of assumptions will improve the technology
4	Climate action	Most disaster preparations are based on assumptions, which cannot always arrest the situation	Natural disaster prediction with AI models will assist in adequate preparation, reducing the losses and providing required remediation planning
5	Climate-smart agriculture	This policy still suffers from several challenges due to inaccurate predictions using previous knowledge	Applying appropriate AI models with weather data to predict climate behavior at different times of the year will support smart agriculture and achieve its potential
6	Carbon dioxide capturing and storage	The system has not achieved the set target due to inadequate planning	Forecasting the amount of CO_2_ expected from time to time using AI will allow adequate planning with little or no CO_2_ escaping into the environment

The accurate and nuanced predictions generated by AI models provide policymakers with a robust foundation for formulating effective climate policies (Srivastava et al. 2023). By understanding the specific impacts on temperature patterns, precipitation regimes, sea level rise, and extreme weather events, policymakers can design targeted interventions that address the most pressing challenges within their jurisdictions. AI models facilitate comprehensive risk assessments by identifying vulnerable areas and populations. Policymakers can leverage this information to prioritize areas at higher risk of climate impacts and implement mitigation measures such as improved infrastructure, early warning systems, and land-use planning to enhance resilience (Rezvani et al. 2023). AI-driven predictions enable the optimization of climate adaptation policies. For example, knowing how precipitation patterns are expected to change allows policymakers to tailor water management strategies, implement drought-resistant agriculture, and enhance flood control measures. This optimization ensures that adaptation efforts are targeted, cost-effective, and aligned with the specific challenges posed by climate change (Blackwood et al. 2022).

AI models contribute to more efficient resource allocation by identifying areas and sectors most susceptible to climate impacts. Whether allocating funds for infrastructure upgrades, disaster preparedness, or conservation efforts, policymakers can prioritize investments based on AI-driven predictions, maximizing the impact of limited resources. Its dynamic and evolving nature characterizes climate change. With their ability to adapt to changing patterns, AI models provide decision-makers with a tool to adjust adaptation strategies dynamically in response to real-time data (Cheong et al. 2022). This flexibility is crucial in the face of unexpected climate events and ensures that adaptation plans remain relevant and effective over time. The implications of climate change on public health are profound, with increased risks of heat-related illnesses, vector-borne diseases, and impacts on air and water quality. AI models can help forecast these health-related impacts, allowing for the development of targeted public health interventions, resource allocation for medical services, and the formulation of policies to protect vulnerable populations (Olawade et al. 2023).

AI-driven predictions seamlessly integrate climate considerations into sustainable development planning (Kulkov et al. 2023). By forecasting the impacts on ecosystems, agriculture, and human communities, planners can align development projects with climate resilience goals, avoiding investments that may become vulnerable to future climate changes. Sustainable development involves finding a balance between economic growth and environmental conservation. AI models can assist in identifying sustainable pathways by assessing the ecological impacts of different development scenarios (Rane et al. 2023). This information enables policymakers to make decisions that foster economic growth while minimizing negative consequences for ecosystems. AI predictions offer insights into the long-term impacts of climate change on infrastructure. Whether designing resilient buildings, planning transportation systems that account for changing weather patterns or developing energy infrastructure that aligns with future climate conditions, AI contributes to sustainable development by informing infrastructure planning.

In conclusion, the implications of AI-driven predictions for policy and decision-making in the context of climate change are far-reaching. From informing policy decisions and optimizing resource allocation to facilitating dynamic adaptation planning and integrating climate considerations into sustainable development, AI models provide a powerful tool for addressing the challenges posed by a changing climate. By incorporating these predictions into decision-making processes, policymakers can create resilient and sustainable strategies that effectively mitigate the impacts of climate change and promote the well-being of both ecosystems and human communities (Hurlbert et al. 2019).

## Challenges and limitations to the adoption of AI model in policy-making and practical climate adaptation strategies

Despite the significant potential shown by AI applications, several challenges hinder the realization of their full benefits. One of the major challenges that limit the adoption of AI in policy-making and practical adaptation strategies is the need for reliance on the high-quality and vast size of data sets. Large volumes of representative and accurate data are required to train models effectively, and the availability of these data may be a serious challenge. Furthermore, careful data curation and preprocessing are essential since data biases can lead to skewed predictions and reinforce preexisting inequities (Olawade et al. 2024). It is still very difficult to scale AI from pilot projects to wider applications. Standardizing AI techniques and tools throughout the different stages while permitting customization to satisfy various level requirements is essential to guaranteeing scalability (Mienye et al. 2024). AI application in policy-making for climate adaptation requires a coherent strategy, which is missing. For easy adoption of AI in climate policy and adaptation, there is a need to develop a comprehensive strategic plan that agrees with AI initiatives with broader application objectives. The expected strategy must identify the goals, performance metrics, and approach for ongoing evaluation and adaptation. Another limitation is resource intensity since advanced AI model deployment and training need significant computer power. Developing nations may find this need prohibitive, limiting their capacity to utilize AI technologies in climate adaptation strategy fully. Due to the possibility that AI models would reinforce biases found in training data, ethical issues also offer serious difficulties. In situations where equal outcomes are critical, ensuring fairness and handling ethical considerations is imperative (Booyse and Scheepers 2024). There may be substantial technological difficulties when integrating AI with antiquated legacy systems. Nevertheless, problems can be overcome by using middleware and APIs strategically, which makes the integration process go more smoothly and gradually. Using this strategy, decision-makers and scientists can take advantage of AI’s advantages without completely revamping their IT infrastructure, which would be expensive and disruptive. AI projects can be severely hampered by a country’s culture that is anti-innovation. Embracing the advantages of AI and promoting innovation requires a culture that embraces experimentation and accepts failure. The nation’s overall ability to undergo digital transformation can be improved by empowering staff members to take the initiative and experiment with new concepts (Jain et al. 2023). The strengths, weaknesses, opportunities, and threats (SWOT) analysis of AI applications in climate change are presented in Table 4. It can be observed from Table 4 that AI can play a significant role in climate change study and decision-making. However, there are still some areas that need improvement to fully annex the potential of AI in climate modeling.

**Table 4:** SWOT analysis of the AI application in climate change modeling

Strengths	Weaknesses
• These technologies can analyze large, intricate datasets while considering several aspects simultaneously. This all-encompassing method enables a more thorough evaluation of ecosystems’ interconnectedness. It aids researchers and decision-makers in comprehending how modifications to one component may impact the entire system • It assists in making better emergency or disaster recovery decisions • Scientists, policymakers, and communities can simulate and forecast future climatic scenarios using predictive models essential to studying climate change • Climate management is aided by optimization and sequential decision-making algorithms to provide a sustainable environment • Predictive AI can be used remotely to support developing and disadvantaged nations against climate phenomena	• Emergency agencies find it challenging to defend choices against disasters using black-box AI algorithms • Climate forecasting requires real-time, accurate information, which is not always affordable • One major issue in the study of climate change is still the interpretability of AI models • Insufficient information to quantify climate change in developing nations • Economic expense and political opposition to using large-scale AI systems to predict urban pollution emissions
Opportunities	Threats
• Early forecasting of natural disasters from climate allows for rapid response by the authorities and minimizes losses • Rainfall forecasting in desert regions enables a better understanding of desertification trends • AI-powered early warning systems to provide a quick and life-saving response in cases of severe famine • Predicting traffic and energy requirements with AI reduces pollutants with a significant ecological impact • AI tools assist in government decision-making in the fight against climate change	• In production, sustainability and cost reduction are frequently mutually exclusive objectives. Communities, scientists, and policymakers may find the expense of incorporating AI into climate change action intolerable • The diversity of AI approaches makes it difficult to select the best methods, especially considering the dearth of personnel with joint expertise in AI and climate change • Unexpected events that impact energy use could make demand forecasts less accurate • The computing cost of AI in terms of climate change impacts is fundamentally high and energy intensive • AI models that use data to predict natural disasters may become outdated due to climate change

## Recommendation and conclusion

This study shows how Artificial Intelligence (AI) can change the game for predicting climate change effects. We can make better predictions by using smart AI methods like machine learning. This helps us understand important issues like rising temperatures, changes in rainfall, sea level rise, and severe weather. AI can analyze huge amounts of data. This helps find patterns that older models might miss. With this information, we can make more accurate assessments of climate impacts. It also helps decision-makers plan better by providing detailed predictions. We must ensure these AI methods are easy for everyone to understand. This way, policymakers and the public can all get the information they need. Teaming up with AI experts, climate scientists, and others makes these models work even better. This helps with planning, resource use, and developing smarter strategies for sustainability.

Looking ahead, we should focus on making our models easier to understand and improve how we deal with uncertainty. It is also important to create AI models that can adjust as climate conditions change. We need to think about fairness and avoid bias so that all communities can adapt to climate change fairly. Plus, we should engage the public to help them understand AI predictions. This builds trust and gets more people involved in decision-making. In summary, using AI in climate science offers great chances to tackle the tough problems of climate change. Ongoing research and teamwork are key to using AI effectively to protect our planet and its people. We can make real progress in fighting climate change by following these suggestions.

## Data Availability

The data is contained within the article and presented in tables and figures.
